# Mild HIE and therapeutic hypothermia: gaps in knowledge with under-powered trials

**DOI:** 10.1038/s41390-024-03537-1

**Published:** 2024-09-17

**Authors:** Lina F. Chalak, Donna M. Ferriero, Alistair J. Gunn, Nicola J. Robertson, Geraldine B. Boylan, Eleanor J. Molloy, Marianne Thoresen, Terrie E. Inder

**Affiliations:** 1 Division of Neonatal-Perinatal Medicine, Department of Pediatrics, University of Texas Southwestern Medical Center, Dallas, TX USA; 2 Department of Neurology, University of California San Francisco, San Francisco, CA USA; 3 Department of Physiology, The University of Auckland, Auckland, New Zealand; 4 Center for Clinical Brain Sciences, University of Edinburgh, Edinburgh, UK; 5 INFANT Research Centre, University College Cork, Cork, Ireland; 6 Discipline of Paediatrics, Trinity College Dublin, The University of Dublin, Dublin, Ireland; 7 St Michael’s Hospital, Child Health, Level D, Bristol, UK; 8 Children’s Hospital of Orange County, Orange, CA USA

The publication of a recent pilot randomized clinical trial (RCT) that investigated whole-body hypothermia (48 or 72 h) as a therapeutic intervention^1^ for infants with mild hypoxic-ischemic encephalopathy (HIE), has prompted experts in trial design in therapeutic hypothermia (TH) to consider opportunities to better understand this trial and the implications for future study designs. This study included 101 patients across six sites (UK and Italy) and aimed to examine whether term infants with mild encephalopathy undergoing TH for 72 h, starting within 6 h, had higher (better) thalamic 1H-magnetic resonance spectroscopy (MRS) N-acetyl aspartate (NAA) concentrations (examined on day 4–14 as a proxy for clinical outcomes) than those who were randomized to standard care at targeted normothermia. The second question in the study compared outcomes related to the length of therapeutic hypothermia (48 or 72 h) for infants randomized after 6 h of age.

HIE presents clinically as neonatal encephalopathy (NE) and affects 2 million infants annually worldwide resulting in a global burden of 50.2 million disability-adjusted life years.^2^ A major challenge in the investigation of mild HIE concerns the accurate definition of mild HIE and the subjective nature of the evaluative clinical examination tools, alongside the need to reach this definition within the therapeutic window of 6 h, despite the evolution of NE over time. This is further confounded by heterogeneity in the timing of the insult.^2^ Contrary to expectations, the study reported that whole-body hypothermia was not associated with better cerebral MRS biomarkers in term born infants with mild HIE compared to normothermia. There are several factors that must be considered in the interpretations of this finding.

Firstly, the patient populations did not appear to be well balanced; infants undergoing hypothermia required more resuscitation, as shown by the much higher number of infants who were intubated at birth in the TH groups (48 h group 48%, 72 h group 36%, normothermia 9%) and more abnormal aEEG at baseline. In addition, normal amplitude on a single channel aEEG performed for at least 30 min between 1 and 6 h of age was one of the criteria for recruitment to the study. Despite this, very little detail is provided about the aEEG recordings, how long they continued and how they were interpreted, though all are reported to show ‘normal aEEG findings’ within 6 h. The authors state that ‘*although all neonates had mild encephalopathy and normal aEEG findings, baseline severity was higher among neonates randomized after vs. before 6* *h of age’*. It is not clear what higher baseline severity means but it does appear to indicate a more abnormal baseline. As most infants in the 48-h and 72-h group were randomized late, findings suggest that these infants may have had more abnormal aEEGs. ‘Electroclinical’ seizures were seen after 6 h of age in 4 infants. However, as only 1 channel aEEG is described, some seizures may have been missed. The gold standard for the detection of seizures in infants is continuous multichannel EEG monitoring (especially in an RCT). The statement ‘*Occurrence of seizures 6* *h after birth was not different across groups*’ needs to be considered in light of this limitation.

Secondly, it is notable that the only major injury seen on magnetic resonance imaging on day 5–7 in all groups was white matter injury, with no deep nuclear gray matter lesions and minimal cortical injury. The timing of the MRI also differed between the groups; the 72-h TH group had the latest average time to MRI. This would allow easier detection of conventional mild abnormalities in the white matter. It would be optimal if the MRI were undertaken at day 4–5 OR after day 7 at day 8–10 of life in all infants in these studies to allow comparable evolution of the diffusion and conventional findings. This can otherwise result in considerable bias in the detection of abnormalities. In addition, the finding of a predominance of white matter injury in these infants raises a comparison to the findings of the large, well designed HELIX trial^3^ that also failed to show any benefit of TH with a predominance of white matter injury that was suggestive of a more subacute inflammatory mediated insult. It is possible that infants with clinical presentations of mild HIE may have suffered a more prolonged subacute intermittent hypoxia-ischemia before, and/or in labor^4^ with secondary energy failure present at birth limiting the effectiveness of therapeutic hypothermia. Against this hypothesis, Table 1 shows that the rates of sentinel events were high and comparable to trials of moderate/severe HIE in high income countries (NT 66.7% vs. 48 h 53% and 72 h 61%).

**Table 1:** Flow Chart Detailing the double randomization for 101 infants.^1^

<6 h Randomization		>6 h Randomization		
NT	HT	NT	HT 48 h	HT72h
34	14	0	31	22

Thirdly and importantly, the study was underpowered. It is notable that only 14 infants who received TH were randomized before 6 h (Table 1). In addition, power was reduced with two levels of randomization, first for early randomization within 6 h to either normothermia or TH for 72 h and then for infants who had already been started on TH before admission, randomization to cooling for either 48 or 72 h. This fragmented the study leading to 2 of the 3 TH groups not being randomized against normothermia. This may have contributed to the apparent greater severity of hypoxia-ischemia in the TH groups. It is not an optimal design to study the effectiveness of duration of TH with multiple subgroups and many infants who were not randomized against normothermia. Further, it is not documented in the paper exactly when TH was started in the late randomization groups. With a standard two group comparison, the p-value would be not significant with numbers provided within 6 h. (Table 1).

Fourthly, although basal ganglia and thalamus (BGT) NAA concentrations and Lactate/NAA peak area ratios are robust biomarkers for 2 year outcomes in moderate and severe HIE,^5^ these MRS biomarkers have not been validated for mild HIE to date and are poorly rated by stakeholders.^6^ The paper did not find improved MRS biomarkers in the cooled group. White matter injury is typical of mild HIE,^7^ yet the authors used thalamic MRS to assess response to therapy, which was not justified. This propensity to white matter injury in mild HIE is confirmed in the published table 2 of Lally et al., using Rutherford’s regional scoring, as noted above, with only white matter injury of different degrees in one third of the patients. Moreover, NAA concentrations increase with increasing gestation and maturity; a GA difference of 3 weeks between 37 and 40 weeks would therefore result in a difference to NAA concentrations. The authors do not mention if they adjusted for this as the normothermic infants have a slightly higher GA.

Finally, neurobehavioral outcomes are not available or reported in these findings, such as the Hammersmith infant neurological examination or importantly 18–24 month neurodevelopmental outcomes. This is the minimal determinant in all trials of TH to assess whether the therapy was effective, and ideally should include school age outcomes.

With consideration of the important information that we have garnered in the effective translation of TH from animal studies, it is noteworthy that TH has been shown to be most effective in the setting of lesser duration of hypoxic-ischemic injury in animal models of HIE. This is seen by the observation in animal models of HIE that the greater the severity of HIE then the more rapid the evolution of cell death. For example, in P21 rat pups (toddler age in humans), after 60 min of hypoxia-ischemia, some cells showed DNA degradation by 10 h after hypoxia, with cortical necrosis at 24 h, whereas after 15 min of hypoxia-ischemia selective neuronal apoptosis only developed after 3 to 5 days.^8^ Consistent with this observation, TH is more protective after milder hypoxia-ischemia at P7.^9^ Similarly, in 7day old rat pups, TH was found to be neuroprotective up to 3 h after 90 min of hypoxia-ischemia but not after more prolonged HIE.^10^ These findings are consistent with data in adult rodents. For example, early initiation of cooling within 1 h and continued for 12 to 24 h after brief ischemia in adult rodents substantially reduced CA1 necrosis at 10 and 30 days, and improved learning in an open field and T-maze after 6 months recovery, with loss of protection if the duration of ischemia was increased.^11,12^ In summary, these animal studies suggest a greater response to hypothermia after less severe insults.

## Take home message

Although infants in the hypothermia groups were sicker at baseline, had more abnormal EEG at baseline and possibly had more seizures, the authors of COMET suggest caution against offering whole-body hypothermia for neonates with mild HIE outside the context of an RCT. While the current study did not demonstrate a significant benefit of whole-body hypothermia in infants with mild HIE as measured by the MRS biomarker thalamic NAA concentrations, the study was underpowered and unbalanced due to its design, which limits its ability to reach a conclusion.

This pilot trial,^1^ albeit with the limitations discussed above, nevertheless serves as a catalyst for future research in neonatal medicine and highlights the complexity of mild HIE, which may well include infants with subacute exposure to hypoxia-ischemia who might not benefit from TH. This underscores the necessity for meticulously designed effectiveness real world randomized trials aimed at elucidating the impact of TH on neurodevelopmental outcomes following mild HIE. By conducting comparative effectiveness studies, researchers can evaluate the benefits and drawbacks of different interventions, helping clinicians make evidence-based decisions regarding patient care.^13^ Such ongoing trials are imperative for advancing our understanding of therapeutic interventions in neonatal care and improving patient outcomes.

## References

[1] Montaldo, P. et al. Whole-body hypothermia vs targeted normothermia for neonates with mild encephalopathy: a multicenter pilot randomized clinical trial. *JAMA Netw. Open* **7**, e249119 (2024). 38709535 10.1001/jamanetworkopen.2024.9119 PMC11074808

[2] Shipley, L., Gale, C. & Sharkey, D. Trends in the Incidence and management of hypoxic-ischaemic encephalopathy in the therapeutic hypothermia era: a national population study. *Arch. Dis. Child Fetal Neonatal Ed.* **106**, 529–534 (2021). 33685945 10.1136/archdischild-2020-320902

[3] Thayyil, S. & Shankaran, S. Rise and fall of therapeutic hypothermia in low-resource settings: lessons from the helix trial: Authors’ reply. *Indian J. Pediatr.* **89**, 311–313 (2022). 34767188 10.1007/s12098-021-03980-6

[4] Burgod, C. et al. Effect of intra-partum oxytocin on neonatal encephalopathy: a systematic review and meta-analysis. *BMC Pregnancy Childbirth* **21**, 736 (2021). 34717571 10.1186/s12884-021-04216-3 PMC8556930

[5] Lally, P. J. et al. Magnetic resonance spectroscopy assessment of brain injury after moderate hypothermia in neonatal encephalopathy: a prospective multicentre cohort study. *Lancet Neurol.* **18**, 35–45 (2019). 30447969 10.1016/S1474-4422(18)30325-9 PMC6291458

[6] Chalak, L., Pilon, B., Byrne, R. & Maitre, N. Stakeholder engagement in neonatal clinical trials: an opportunity for mild neonatal encephalopathy research. *Pediatr. Res.* **93**, 4–6 (2023). 35477747 10.1038/s41390-022-02067-y

[7] Li, Y. et al. Mild Hypoxic-Ischemic Encephalopathy (Hie): timing and pattern of Mri brain injury. *Pediatr. Res.* **92**, 1731–1736 (2022). 35354930 10.1038/s41390-022-02026-7 PMC9771796

[8] Beilharz, E. J., Williams, C. E., Dragunow, M., Sirimanne, E. S. & Gluckman, P. D. Mechanisms of delayed cell death following hypoxic-ischemic injury in the immature rat: evidence for apoptosis during selective neuronal loss. *Brain Res. Mol. Brain Res.* **29**, 1–14 (1995). 7769986 10.1016/0169-328x(94)00217-3

[9] Wood, T. et al. Treatment temperature and insult severity influence the neuroprotective effects of therapeutic hypothermia. *Sci. Rep.* **6**, 23430 (2016). 26997257 10.1038/srep23430 PMC4800445

[10] Sabir, H., Scull-Brown, E., Liu, X. & Thoresen, M. Immediate hypothermia is not neuroprotective after severe hypoxia-ischemia and is deleterious when delayed by 12 h in neonatal rats. *Stroke* **43**, 3364–3370 (2012). 22996953 10.1161/STROKEAHA.112.674481

[11] Colbourne, F. & Corbett, D. Delayed and prolonged post-ischemic hypothermia is neuroprotective in the Gerbil. *Brain Res.* **654**, 265–272 (1994). 7987676 10.1016/0006-8993(94)90488-x

[12] Colbourne, F. & Corbett, D. Delayed postischemic hypothermia: a six month survival study using behavioral and histological assessments of neuroprotection. *J. Neurosci.* **15**, 7250–7260 (1995). 7472479 10.1523/JNEUROSCI.15-11-07250.1995 PMC6578099

[13] Chalak, L. New horizons in mild hypoxic-ischemic encephalopathy: a standardized algorithm to move past conundrum of care. *Clin. Perinatol.* **49**, 279–294 (2022). 35210007 10.1016/j.clp.2021.11.016

